# Refining histopathological growth pattern-based risk group discrimination in nodular lymphocyte-predominant Hodgkin lymphoma: an analysis from the German Hodgkin Study Group

**DOI:** 10.1038/s41375-025-02641-3

**Published:** 2025-05-13

**Authors:** Dennis A. Eichenauer, Aylin Basaran, Ina Bühnen, Michael Fuchs, Bastian von Tresckow, Andreas Rosenwald, Martin-Leo Hansmann, Heinz-Wolfram Bernd, Peter Borchmann, Wolfram Klapper, Sylvia Hartmann

**Affiliations:** 1 University of Cologne, First Department of Internal Medicine, Center for Integrated Oncology Aachen Bonn Cologne Dusseldorf, Cologne, Germany; 2 German Hodgkin Study Group (GHSG), University Hospital Cologne, Cologne, Germany; 3 Department of Hematology and Stem Cell Transplantation, University Hospital Essen, University Duisburg-Essen, German Cancer Consortium (DKTK partner site Essen), Essen, Germany; 4 Institute of Pathology, University of Würzburg and Comprehensive Cancer Center (CCC) Mainfranken, Würzburg, Germany; 5 Institute of Pathology and Molecular Pathology, Helios University Hospital Wuppertal, Wuppertal, Germany; 6 Hematopathology Lübeck, Lübeck, Germany; 7 Institute of Pathology, Hematopathology Section and Lymph Node Registry, University Hospital Schleswig-Holstein Campus Kiel, Kiel, Germany; 8 Institute of Pathology, University Hospital Essen, University Duisburg-Essen, Essen, Germany

**Keywords:** Hodgkin lymphoma, Hodgkin lymphoma

## Abstract

Histopathological growth patterns (GP) in nodular lymphocyte-predominant Hodgkin lymphoma (NLPHL) have previously been divided into GP AB (typical) vs CDEF (variant). However, it is unclear whether this division is optimal. We thus investigated alternative GP grouping approaches (GP ABC vs DEF; GP ABCF vs DE). Overall, 583 NLPHL patients who had first-line treatment within GHSG trials were included in the analysis. Median age was 39 years; 74% of patients were male; 76% presented with early-stage and 24% with advanced-stage disease. The 5-year and 10-year progression-free survival (PFS) estimates for all patients were 85.9% and 76.6%; overall survival (OS) estimates were 95.8% and 94.5%. Significant PFS and OS differences were detected for the comparison GP ABCF vs DE with worse outcomes for the GP DE group (HR: 1.7; 95%-CI: 1.1–2.7; HR: 2.5; 95%-CI: 1.1–5.7). No PFS and OS differences were observed for the comparisons GP AB vs CDEF and GP ABC vs DEF. Median time to death was shorter and death more often due to NLPHL in the GP DE (13 months; 66.7%) than in the GP ABCF (31 months; 5.6%) group. Hence, the division of GP into GP ABCF vs DE allows an optimized GP-based risk group discrimination in NLPHL.

## Introduction

Nodular lymphocyte-predominant Hodgkin lymphoma (NLPHL) accounts for roughly 5% of all Hodgkin lymphoma (HL) cases. Most NLPHL patients are diagnosed in early stages and the clinical course is usually indolent [1]. Excess mortality in comparison with the general population is low [2]. In contrast to classical HL (cHL), the disease-defining lymphocyte predominant (LP) cells in NLPHL are consistently positive for CD20 and typically negative for CD30 [1].

**Fig. 1:**
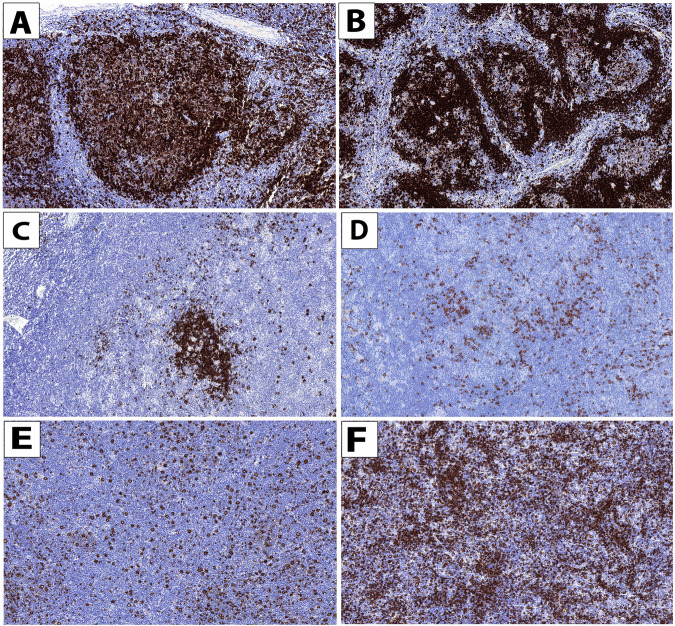
Histopathological growth patterns. Histopathological growth patterns (GP) according to Fan et al. [3]; CD20 immunostaining (sixfold magnification): GP A (classic nodular) (**A**), GP B (serpiginous/interconnected nodular) (**B**), GP C (prominent extranodular LP cells) (**C**), GP D (T-cell-rich nodular) (**D**), GP E (T-cell-rich large B-cell-like) (**E**), GP F (diffuse B-cell-rich) (**F**).

Based on the localization of the LP cells in the tumor tissue and the composition of the microenvironment, six histopathological growth patterns (GP) have been described in NLPHL (Fig. 1) [3]. The GP A and B (AB) are often termed typical whereas the GP CDEF are considered as variants. Several studies have demonstrated that the GP have some prognostic impact when divided into GP AB vs CDEF. An analysis from the German Hodgkin Study Group (GHSG) included 413 NLPHL patients who had received first-line treatment within prospective trials. At 5 years, patients with GP AB had a better progression-free survival (PFS) than individuals with GP CDEF [4]. A retrospective analysis from the International Lymphoma Radiation Oncology Group (ILROG) including individuals with stage I/II NLPHL also indicated a better PFS for patients with GP AB than for individuals with GP CDEF if treatment consisted of radiotherapy (RT) alone [5]. However, it is unclear whether the division of GP into GP AB vs CDEF is optimal. In the International Consensus Classification (ICC), the Clinical Advisory Committee (CAC) has suggested to distinguish GP ABC (grade I) from GP DEF (grade II) although data supporting this grouping approach are not available until now [6]. Another alternative might consist in the grouping of GP into those with a higher B-cell content in the microenvironment (GP ABCF) on one hand and those with a higher T-cell content (GP DE) on the other hand. In early-stage unfavorable cHL, it has been demonstrated that a low B-cell content in the tumor microenvironment is associated with an impaired PFS [7].

To gain in-depth insight into the association between GP and patient characteristics, course, PFS, second-line treatment, overall survival (OS) and causes of death and to determine the GP grouping approach allowing for the best discrimination in terms of survival outcomes (GP AB vs CDEF, GP ABC vs DEF or GP ABCF vs DE), we performed an analysis comprising patients who had treatment for newly diagnosed NLPHL within 12 prospective GHSG trials conducted between 1998 and 2017.

## Subjects and methods

### Patients and treatment

Patients with NLPHL (as confirmed by expert review) and available information on the GP at initial diagnosis (as determined by expert review) who had first-line treatment within the GHSG LP, LPHD and RIPL studies for stage IA disease without risk factors (large mediastinal mass, extranodal disease, elevated erythrocyte sedimentation rate, involvement of 3 or more nodal areas), the HD10, HD13 and HD16 studies for early-stage favorable disease (stage I/II without risk factors), the HD11, HD14 and HD17 studies for early-stage unfavorable disease (stage I/IIA with one or more of the risk factors large mediastinal mass, extranodal disease, elevated erythrocyte sedimentation rate and involvement of 3 or more nodal areas; stage IIB with one or both of the risk factors elevated erythrocyte sedimentation rate and involvement of 3 or more nodal areas) and the HD12, HD15 and HD18 studies for advanced-stage disease (stage IIB with one or both of the risk factors large mediastinal mass and extranodal disease; stage III/IV) were eligible for the present analysis. Study treatment consisted of RT alone, single-agent anti-CD20 antibody treatment with rituximab, chemotherapy alone, chemotherapy plus consolidation RT and chemotherapy plus rituximab optionally followed by consolidation RT, respectively. The applied chemotherapy protocols were ABVD (doxorubicin, bleomycin, vinblastine, dacarbazine), ABVD-like regimens and different BEACOPP (bleomycin, etoposide, doxorubicin, cyclophosphamide, vincristine, procarbazine, prednisone) variants. Details regarding the treatment within the studies have been described elsewhere [8–12]. The studies were approved by the review boards of the participating sites and were conducted in accordance with the Declaration of Helsinki. Informed consent was obtained from all study participants.

### Statistical methods

Comparisons of patient characteristics and outcomes according to the GP groups (in patients with more than one detected GP, the order of relevance for the individual GP was as follows: AB < C < D < E < F; as an example, pattern E was considered as relevant GP if GP C and E had been detected) were conducted using Fisher’s exact test and log-rank test as applicable. Additional analyses were performed descriptively. PFS was defined as time from completion of initial staging until disease progression or death from any cause. If none of these events had occurred, PFS was censored at the date of last information on the disease status. Histological transformation into aggressive B-cell non-Hodgkin lymphoma (B-NHL) was not considered as PFS event. OS was defined as time from completion of initial staging until death from any cause and was censored at the date of last information for surviving patients. Time-to-event endpoints were investigated using the Kaplan-Meier method and Cox regression analyses including hazard ratios (HR) and 95% confidence intervals (95%-CI). SAS version 9.4 for Microsoft Windows (SAS Institute, Cary, NC) was used for all analyses.

## Results

### Patient characteristics

A total of 583 NLPHL patients were included in the present analysis. The median age was 39 years (range: 19–75 years). Most patients were male (433/583 patients; 74.3%). At diagnosis, 168/583 patients (28.8%) had stage IA disease without risk factors, 206/583 (35.3%) early favorable stages other than stage IA without risk factors, 69/583 (11.8%) early unfavorable stages and 140/583 (24%) advanced stages (Table 1). Information on individual GP was available for 581 patients; for 2 patients, GP CDEF without specification of individual GP were documented. Of those with available information on individual GP, 408 (70.2%) had GP AB, 60 (10.3%) GP C, 68 GP D (11.7%), 30 GP E (5.2%) and 15 GP F (2.6%). The proportion of GP AB was higher in patients with stage IA disease without risk factors (134/168 patients; 79.8%), early favorable stages other than stage IA without risk factors (149/204 patients; 73%) and early unfavorable stages (53/69 patients; 76.8%) than in individuals with advanced stages (72/140 patients; 51.4%). Conversely, especially the GP D and E were more common in patients with advanced stages (GP D: 35/140 patients; 25%; GP E: 15/140 patients; 10.7%) than in individuals with stage IA disease without risk factors (GP D: 9/168 patients; 4.5%; GP E: 2/168 patients; 1.2%), early favorable stages other than stage IA without risk factors (GP D: 20/204 patients; 9.8%; GP E: 10/204 patients; 4.9%) and early unfavorable stages (GP D: 4/69 patients; 5.8%; GP E: 3/69 patients; 4.2%) (Supplementary Table 1). When grouping and comparing patients according to their GP (GP AB vs CDEF; GP ABC vs DEF; GP ABCF vs DE), the distribution of risk groups according to the GHSG criteria, the presence of B symptoms as well as the frequency of splenic, liver and bone marrow involvement differed significantly between the groups for all three comparisons (Table 1).

**Table 1:** Patient characteristics.

		AB (*N* = 408)	CDEF (*N* = 175)	*P* value	ABC (*N* = 468)^a^	DEF (*N* = 113)^a^	*P* value	ABCF (*N* = 483)^a^	DE (*N* = 98)^a^	P-value	Total (*N* = 583)
N (%)											
**Age in years**	**median (min-max)**	40 (16–75)	38.5 (16–71)	0.2552	39 (16–75)	39 (16–71)	0.5777	39 (16–75)	39 (16–71)	0.3665	39 (16–75)
**Sex**	**Female**	112 (27.5)	38 (21.7)	0.1788	124 (26.5)	26 (23)	0.4749	128 (26.5)	22 (22.4)	0.4490	150 (25.7)
	**Male**	296 (72.5)	137 (78.3)		344 (73.5)	87 (77)		355 (73.5)	76 (77.6)		433 (74.3)
**Risk group according to GHSG criteria**	**Stage IA w/o RF**^b^	134 (32.8)	34 (19.4)	<0.0001	152 (32.5)	16 (14.2)	<0.0001	157 (32.5)	11 (11.2)	<0.0001	168 (28.8)
	**Early favorable**	149 (36.5)	57 (32.6)		169 (36.1)	35 (31)		174 (36)	30 (30.6)		206 (35.3)
	**Early unfavorable**	53 (13)	16 (9.1)		60 (12.8)	9 (8)		62 (12.8)	7 (7.1)		69 (11.8)
	**Advanced**	72 (17.6)	68 (38.9)		87 (18.6)	53 (46.9)		90 (18.6)	50 (51)		140 (24)
**B symptoms**	**Yes**	34 (8.3)	29 (16.6)	0.0053	39 (8.3)	24 (21.2)	0.0003	41 (8.5)	22 (22.4)	0.0002	63 (10.8)
**Organ involvement**^c^	**Spleen**	20/394 (5.1)	36/173 (20.8)	<0.0001	24/452 (5.3)	32/113 (28.3)	<0.0001	24/467 (5.1)	32/98 (32.7)	<0.0001	56/567 (9.9)
	**Liver**	3/397 (0.8)	9/174 (5.2)	0.0017	4/456 (0.9)	8/113 (7.1)	0.0005	4/471 (0.8)	8/98 (8.2)	0.0002	12/571 (2.1)
	**Bone marrow**	3/397 (0.8)	13/174 (7.5)	<0.0001	4/456 (0.9)	12/113 (10.6)	<0.0001	6/471 (1.3)	10/98 (10.2)	<0.0001	16/571 (2.8)
	**Extranodal**	17/396 (4.3)	14/174 (8)	0.0740	19/455 (4.2)	12/113 (10.6)	0.0178	22/470 (4.7)	9/98 (9.2)	0.0864	31/570 (5.4)
**LP-IPS**^c^	**0–1**	345/384 (89.8)	122/172 (70.9)	<0.0001	393/441 (89.1)	72/113 (63.7)	<0.0001	407/456 (89.3)	58/98 (59.2)	<0.0001	467/556 (84%)
	**2–4**	39/384 (10.2)	50/172 (29.1)		48/441 (10.9)	41/113 (36.3)		49/456 (10.7)	40/98 (40.8)		89/556 (16)

^a^581/583 with relevant pattern documented.

^b^RF: risk factors.

^c^Documentation not available for all patients.

### Progression-free survival

**Fig. 2:**
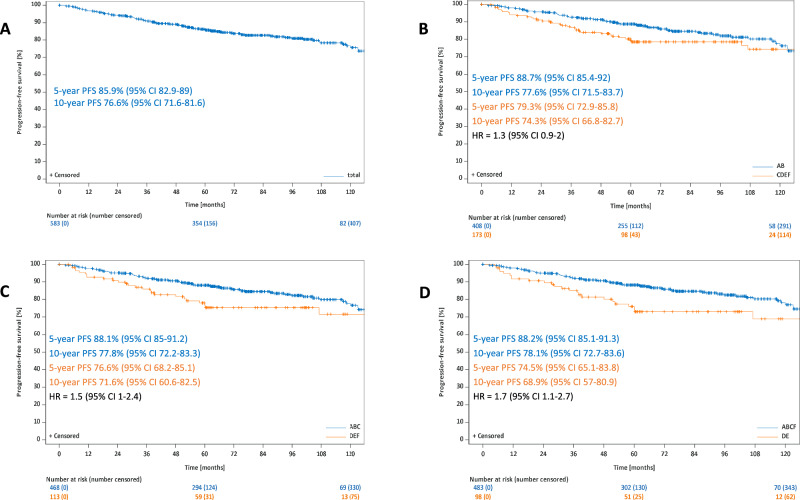
Progression-free survival for all patients. Progression-free survival for all patients (**A**), for the comparison GP AB vs CDEF (**B**), for the comparison GP ABC vs DEF (**C**) and for the comparison ABCF vs DE (**D**).

After a median observation time of 78 months in terms of PFS, 5-year and 10-year estimates for all 583 patients were 85.9% (95%-CI: 82.9–89%) and 76.6% (95%-CI: 71.6–81.6%). Individuals from the GP AB group had 5-year and 10-year PFS rates of 88.7% (95%-CI: 85.4–92%) and 77.6% (95%-CI: 71.5–83.7%) as compared to 79.3% (95%-CI: 72.9–85.8%) and 74.3% (95%-CI: 66.8–82.7%) for patients with GP CDEF (HR: 1.3; 95%-CI: 0.9–2). The 5-year and 10-year PFS rates for patients from the GP ABC group were 88.1% (95%-CI: 85–91.2%) and 77.8% (95%-CI: 72.2–83.3%) whereas patients with GP DEF had 5-year and 10-year PFS rates of 76.6% (95%-CI: 68.2–85.1%) and 71.6% (95%-CI: 60.6–82.5%) (HR: 1.5; 95%-CI: 1–2.4). The GP ABCF group had 5-year and 10-year PFS rates of 88.2% (95%-CI: 85.1–91.3%) and 78.1% (95%-CI: 72.7–83.6%), the respective rates for patients with GP DE were 74.5% (95%-CI: 65.1–83.8%) and 68.9% (57–80.9%) (HR: 1.7; 95%-CI: 1.1–2.7). Hence, a significant PFS difference was only detected for the comparison GP ABCF vs DE (Fig. 2).

**Fig. 3:**
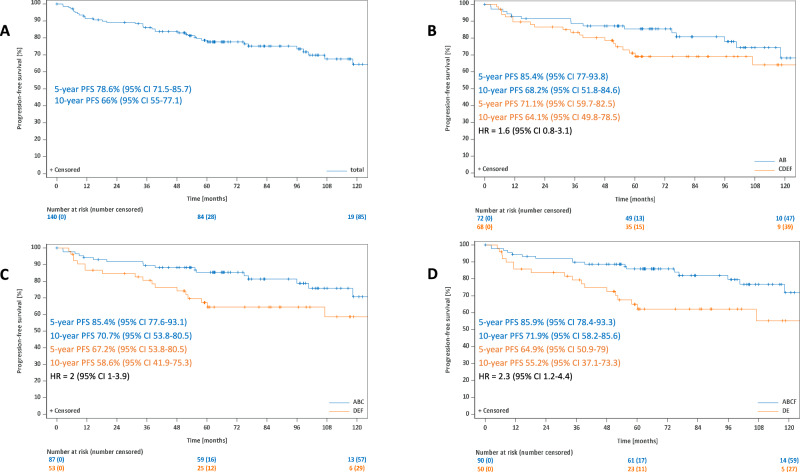
Progression-free survival for patients with advanced-stage disease. Progression-free survival for all patients with advanced stages (**A**), for the comparison GP AB vs CDEF in advanced-stage patients (**B**), for the comparison GP ABC vs DEF in advanced-stage patients (**C**) and for the comparison GP ABCF vs DE in advanced-stage patients (**D**).

If analyses were performed separately for early-stage (stage IA disease without risk factors, early favorable stages other than stage IA without risk factors, early unfavorable stages) (*n* = 443) and advanced-stage disease (*n* = 140), respectively, no PFS difference was observed for any of the three GP group comparisons in early-stage patients (GP AB *vs* CDEF: HR: 1; 95%-CI: 0.5–1.7; GP ABC vs DEF: HR: 0.8; 95%-CI: 0.4–1.4; GP ABCF vs DE: HR: 0.8; 95%-CI: 0.4–2) (Supplementary Fig. 1). For advanced-stage disease, a PFS difference was detected for the comparison GP ABCF *vs* DE (HR: 2.3; 95%-CI: 1.2–4.4) but not for the comparisons GP AB *vs* CDEF (HR: 1.6; 95%-CI: 0.8–3.1) and GP ABC *vs* DEF (HR: 2; 95%-CI: 1–3.9) (Fig. 3).

### Relapse characteristics and second-line treatment

A total of 84 patients included in the present analysis had disease recurrence during follow-up. The median time to relapse was 39 months (range: 3–144 months). Patients with GP AB had a longer median time to relapse than patients with GP CDEF (GP AB: 53 months; GP CDEF: 27 months). Similarly, patients with GP ABC had a longer median time to relapse in comparison with individuals with GP DEF (GP ABC: 49 months; GP DEF: 29 months) and the median time to relapse was longer for patients with GP ABCF as compared to patients with GP DE (GP ABCF: 47 months; GP DE: 30 months).

Information on the second-line treatment was available for 76/84 patients (90.5%) with NLPHL recurrence. Of these, 29 (38.2%) had high-dose chemotherapy and autologous stem cell transplantation (ASCT), 21 (27.6%) conventional chemotherapy optionally combined with an anti-CD20 antibody and/or RT and 23 (31.6%) single-agent anti-CD20 antibody treatment and/or RT; 2 patients (2.6%) had treatment within a prospective trial investigating the Bruton´s tyrosine kinase inhibitor ibrutinib in relapsed NLPHL [13]. Salvage approaches differed according to the GP. The GP AB, GP ABC and GP ABCF groups more often had non-intensive salvage therapy consisting of single-agent anti-CD20 antibody treatment and/or RT (GP AB: 37%; GP ABC: 34.5%; GP ABCF: 35.7%) than the groups with GP CDEF, GP DEF and GP DE (GP CDEF: 23.3%; GP DEF: 23.8%; GP DE: 20%). Conversely, patients from the latter groups more frequently had intensive second-line treatment with high-dose chemotherapy and ASCT (GP CDEF: 50%; GP DEF: 52.4%; GP DE: 55%) than patients from the GP AB (30.4%), GP ABC (32.7%) and GP ABCF (32.1%) groups (Table 2).

**Table 2:** Relapse characteristics and salvage treatment at first relapse.

		AB (*N* = 408)	CDEF (*N* = 175)	*P* value	ABC (*N* = 468)	DEF (*N* = 113)	*P* value	ABCF (*N* = 483)	DE (*N* = 98)	*P* value	Total (*N* = 84)
**Relapse**	**n (%)**	52 (12.7)	32 (18.2)	0.1187	61 (11.9)	23 (20.4)	0.0286	62 (12.8)	22 (22.4)	0.0229	84 (14.4)
**Median number of relapses**	**Median (min-max)**	1 (1–2)	1 (1–4)	0.1187	1 (1–2)	1 (1–4)	0.0286	1 (1–2)	1 (1–4)	0.0229	1 (1–4)
**Median time to first relapse (months)**	**Median (min-max)**	53 (3–144)	27 (5–107)	0.0020	49(3–144)	29 (5–107)	0.0187	47 (3–144)	30 (5–107)	0.0259	39 (3–144)
		**AB (*N* = 52)**	**CDEF (*N* = 32)**		**ABC (*N* = 61)**	**DEF (*N* = 23)**		**ABCF (*N* = 62)**	**DE (*N* = 22)**		**Total (*N* = 84)**
		**n (%)**									
**HDCT**^a^ **and ASCT**^b^		14/46 (30.4)	15/30 (50)		18/55 (32.7)	11/21 (52.4)		18/56 (32.1)	11/20 (55)		29/76 (38.2)
**Conventional chemotherapy**		13/46 (28.3)	8/30 (26.7)		16/55 (29.1)	5/21 (23.8)	5/21 (23.8)	16/56 (28.6)	5/20 (25)		21/76 (27.6)
**Radiotherapy and/or anti-CD20 antibody**		17/46 (37)	7/30 (23.3)		19/55 (34.5)	5/21 (23.8)	5/21 (23.8)	20/56 (35.7)	4/20 (20)		23/76 (31.6)
**Other**^**c**^		2/46 (4.3)	—		2/55 (3.6)	—		2/56 (3.6)	—		2/76 (2.6)

Information on treatment at first relapse not available for 8/84 patients (9.5%).

^a^HDCT: high-dose chemotherapy.

^b^ASCT: autologous stem cell transplantation.

^c^Single-agent ibrutinib.

### Second primary malignancies

Second primary malignancies during follow-up occurred in 38/583 patients (6.5%). Of these, 20 (52.6%) had solid tumors and 18 (47.4%) hematologic malignancies. The median time from NLPHL diagnosis to the occurrence of a second primary malignancy was 37 months (range: 7–178 months) for all cases; it was 44 months (range: 7–178 months) for second solid tumors and 33 months (range: 9–165 months) for second hematologic malignancies (Supplementary Table 2). Second hematologic malignancies included 11 cases of aggressive B-NHL (GP AB: 8/11 cases; GP ABC: 9/11 cases; GP ABCF: 9/11 cases; CDEF: 3/11 cases; GP DEF: 2/11 cases; GP D/E: 2/11 cases). Aggressive B-NHL occurred after a median time of 24 months (range: 9–165 months) from the initial NLPHL diagnosis (not shown).

### Overall survival

**Fig. 4:**
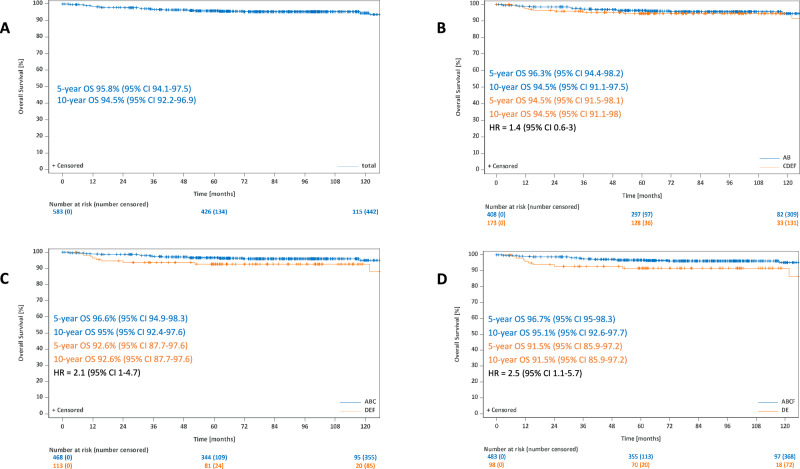
Overall survival for all patients. Overall survival for all patients (**A**), for the comparison GP AB vs CDEF (**B**), for the comparison GP ABC vs DEF (**C**) and for the comparison ABCF vs DE (**D**).

After a median observation time 86 months in terms of OS, the 5-year and 10-year estimates for all patients were 95.8% (95%-CI: 94.1–97.5%) and 94.5% (95%-CI: 92.2–96.9%). The 5-year and 10-year OS rates were 96.3% (95%-CI: 94.4–98.2%) and 94.5% (95%-CI: 91.5–97.5%) for patients with GP AB and 94.5% each (95%-CI: 91.5–98.1% at 5 years and 91.1–98% at 10 years) for individuals with GP CDEF (HR: 1.4; 95%-CI: 0.6–3), 96.6% (95%-CI: 94.9–98.3%) and 95% (95%-CI: 92.4–97.6%) for the GP ABC group and 92.6% each (95%-CI: 87.7–97.6% at 5 and 10 years each) for the GP DEF group (HR: 2.1; 95%-CI: 1–4.7). The GP ABCF group had a 5-year OS rate of 96.7% (95%-CI: 95–98.3%) and a 10-year OS rate of 95.1% (95%-CI: 92.6–97.7%) while individuals from the GP DE group had 5-year and 10-year OS rates of 91.5% each (95%-CI: 85.9–97.2% at 5 and 10 years each) (HR: 2.5; 95%-CI: 1.1–5.7). A significant difference was thus only detected for the comparison GP ABCF vs DE (Fig. 4).

**Fig. 5:**
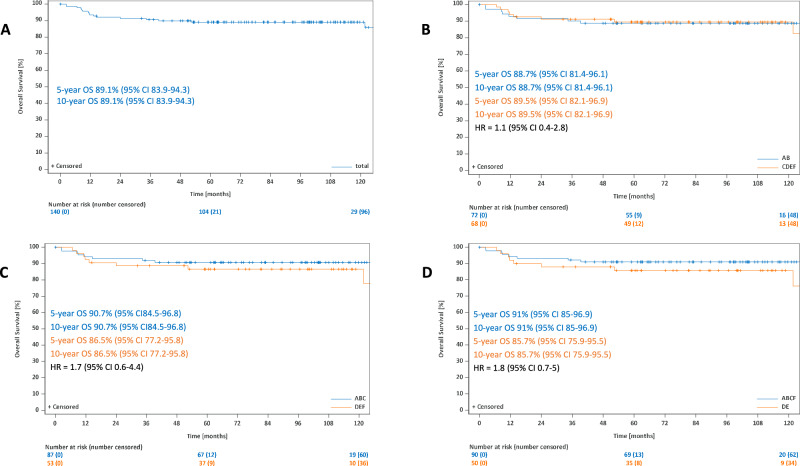
Overall survival for patients with advanced-stage disease. Overall survival for all patients with advanced stages (**A**), for the comparison GP AB vs CDEF in advanced-stage patients (**B**), for the comparison GP ABC vs DEF in advanced-stage patients (**C**) and for the comparison GP ABCF vs DE in advanced-stage patients (**D**).

If separate OS analyses were performed for patients with early-stage disease (stage IA disease without risk factors, early favorable stages other than stage IA without risk factors, early unfavorable stages) and advanced stages, no OS differences were detected in any of the GP group comparisons (early stages: GP AB vs CDEF: HR: 0.7; 95%-CI: 0.2–3.3; GP ABC vs DEF: HR: 0.7; 95%-CI: 0.1–5.2; GP ABCF vs DE: HR: 0.9; 95%-CI: 0.1–6.7; advanced stages: GP AB vs CDEF: HR: 1.1; 95%-CI: 0.4–2.8; GP ABC vs DEF: HR: 1.7; 95%-CI: 0.6–4.4; GP ABCF vs DE: HR: 1.8; 95%-CI: 0.7–5) (Supplementary Fig. 2, Fig. 5).

### Causes of death

Overall, 27 patients died during follow-up. The median time between NLPHL diagnosis and death was 24 months (range: 2–122 months). The median time to death was longer for the GP AB (30 months), GP ABC (31 months) and GP ABCF (31 months) groups than for the GP CDEF (14 months), GP DEF (13 months) and GP DE (13 months) groups. Among all patients who died, the most common causes of death were second primary malignancies (11/27 deaths; 41.4%) and NLPHL (7/27 deaths; 24.1%). However, the distribution of causes of death differed according to the GP. Second primary malignancies represented the major cause of death in the GP AB (9/17 deaths; 52.9%), GP ABC (10/18 deaths; 55.6%) and GP ABCF (10/18 deaths; 55.6%) groups. The most common cause of death for the GP CDEF (6/10 deaths; 60%), GP DEF (6/9 deaths; 66.7%) and GP DE (6/9 deaths; 66.7%) groups was NLPHL which only rarely represented the cause of death in the GP AB (1/17 deaths; 5.9%), GP ABC (1/18 deaths; 5.6%) and GP ABCF (1/18 deaths; 5.6%) groups (Table 3).

**Table 3:** Characteristics of death in patients who died during follow-up.

		AB (*N* = 408)	CDEF (*N* = 175)	ABC (*N* = 468)	DEF (*N* = 113)	ABCF (*N* = 483)	DE (*N* = 98)	Total (*N* = 583)
**n (%)**								
**Deaths**		17/408 (4.2)	10/175 (5.7)	18/468 (43.8)	9/114 (7.9)	18/483 (3.7)	9/98 (9.2)	27/583 (4.6)
	**NLPHL-associated**	1/17 (5.9)	6/10 (60)	1/18 (5.6)	6/9 (66.7)	1/18 (5.6)	6/9 (66.7)	7/27 (24.1)
	**Treatment-associated**	2/17 (11.8)	1/10 (10)	2/18 (11.1)	1/9 (11.1)	2/18 (11.1)	1/9 (11.1)	3/27 (10.3)
	**Second primary malignancy**	9/17 (52.9)	2/10 (20)	10/18 (55.6)	1/9 (11.1)	10/18 (55.6)	1/9 (11.1)	11/27 (41.4)
	**Unknown cause**	1/17 (5.9)	—	1/18 (5.6)	—	1/18 (5.6)	—	1/27 (3.7)
	**Other**	4/17 (23.5)	1/10 (10)	4/18 (22.2)	1/9 (11.1)	4/18 (22.2)	1/9 (11.1)	5/27 (18.5)
	**Median time to death (min-max)**	30 (2–118)	14 (7–122)	31(2–118)	13 (7–122)	31 (2–118)	13 (7–122)	24(2–122)
	**Median age at death in years (min-max)**	53(36–62)	49 (25–73)	53 (36–73)	46 (25–66)	53 (36–73)	46 (25–66)	52 (25–73)

## Discussion

To our knowledge, this is one of the largest reports on the impact of histopathological GP on clinical presentation and course of NLPHL patients. It is also the first study addressing GP grouping approaches other than GP AB vs CDEF. The following major findings evolved from the present analysis: (1) baseline characteristics differ between GP groups for all three conducted GP group comparisons; (2) the division of GP into GP ABCF vs DE results in a better discrimination in terms of PFS and OS than the grouping approaches GP AB vs CDEF and GP ABC vs DEF; (3) relapse characteristics, second-line approaches and causes of death differ between GP groups for all three comparisons.

In the present analysis, the median age among the 583 included patients was 39 years. Males accounted for 74.3% of cases, 76% of patients had early-stage disease at diagnosis. Regarding the histopathological GP, 70.2% of patients presented with GP AB. A population-based analysis using the Surveillance, Epidemiology and End Results (SEER) database included a total of 1162 patients with NLPHL. The median age was 38 years, 69% of patients were male [14]. In a large retrospective analysis comprising data from 2243 NLPHL patients of all ages who had been treated at institutions worldwide, the median age was 37 years, male children and men accounted for 74.9% of cases and 73.8% of patients with available information on the GP had GP AB [15].

In the present analysis, the PFS estimates at 5 and 10 years were 85.9% and 76.6%, respectively. Although PFS rates differed numerically between groups for all three GP group comparisons, a significant PFS difference was only observed for the comparison GP ABCF vs DE (HR: 1.7; 95%-CI: 1.1–2.7). Thus, previously detected 5-year PFS differences between patients with GP AB and individuals with GP CDEF appear to diminish or even disappear with longer follow-up. An earlier analysis from the GHSG including 308 patients with GP AB and 105 patients with GP CDEF had indicated a significantly better PFS at 5 years for individuals with GP AB [4]. This disappearance of a significant PFS difference between patients with GP AB and individuals with GP CDEF with extended follow-up might at least in part be due to different relapse characteristics associated with GP AB and GP CDEF, respectively. A retrospective study comprising 33 NLPHL patients treated within GHSG studies revealed a median time to relapse of 5.2 years for patients with GP AB (*n* = 21) vs 2.8 years for patients with GP CDEF (*n* = 12) (*p* = 0.0219). Thus, disease recurrence beyond 5 years of follow-up occurs in a significant proportion of patients especially in the GP AB group [16]. This finding was confirmed in the present analysis with a median time to relapse of 53 months for GP AB vs 27 months for GP CDEF. Similar results were obtained for the comparisons GP ABC vs DEF (median time to relapse: 49 months vs 29 months) and GP ABCF vs DE (median time to relapse: 47 months vs 30 months).

The PFS difference between patients with GP ABCF and individuals with GP DE was most pronounced in the advanced-stage group (HR: 2.3; 95%-CI: 1.2–4.4). This is remarkable since a large study including 916 NLPHL patients with information on the GP was unable to demonstrate a significant impact of the GP on PFS in multivariable analyses and the coincidence of particularly GP E and advanced-stage disease has been discussed as possible factor contributing to this finding [15].

Second-line treatment among patients from the present analysis who developed NLPHL recurrence consisted of high-dose chemotherapy and ASCT in 38.2% of cases, 27.6% had conventional chemotherapy optionally combined with an anti-CD20 antibody and/or RT and 31.6% anti-CD20 antibody treatment and/or RT; 2.6% of relapses were treated with the Bruton´s tyrosine kinase inhibitor ibrutinib. Hence, the overall distribution of salvage approaches was similar to previous analyses addressing salvage therapy in NLPHL [17, 18]. In the present analysis, the GP AB, GP ABC and GP ABCF groups less frequently had intensive salvage therapy with high-dose chemotherapy and ASCT whereas non-intensive second-line treatment with an anti-CD20 antibody and/or RT was more often applied than in the GP CDEF, GP DEF and GP DE groups. Different factors including less intensive first-line treatment with no or only small amounts of chemotherapy due to limited disease at initial diagnosis and an often more indolent clinical course may have enabled non-intensive second-line treatment without high-dose chemotherapy and ASCT in a high proportion of patients from the GP AB, GP ABC and GP ABCF groups.

The present analysis revealed a significantly better OS for patients with GP ABCF than for patients with GP DE whereas no OS differences were detected for the comparisons GP AB vs CDEF and GP ABC vs DEF. In addition, the median time to death and the causes of death differed between the GP AB, GP ABC and GP ABCF groups and the GP CDEF, GP DEF and GP DE groups. The time interval between the initial NLPHL diagnosis and death was longer and second primary malignancies represented the major cause of death for the GP AB, GP ABC and GP ABCF groups. In contrast, patients from the GP CDEF, GP CDE and GP DE groups mostly died from NLPHL. The detection of an OS difference only for the comparison GP ABCF vs DE indicates that the grouping approach GP ABCF vs DE is superior to the traditional GP grouping, i.e. GP AB vs CDEF and the grouping approach proposed by the ICC, i.e. GP ABC vs DEF [4, 6]. The group differences regarding the causes of death underscore the need to reduce treatment toxicity in low-risk patients to mitigate the risk for the development of potentially fatal late effects such as second primary malignancies while maintaining a sufficient treatment intensity in patients with a higher risk for disease recurrence and NLPHL-related death. A better identification of these patients may be achieved by implementing factors such as the GP and the lymphocyte-predominant international prognostic score (LP-IPS) into risk group allocation systems which are commonly based on the stage according to the Ann Arbor classification and the presence of clinical risk factors. The LP-IPS is based on data from 2243 NLPHL patients and allows to distinguish four risk groups with 5-year PFS rates ranging from 59 to 88.4% and 5-year OS rates ranging from 83.3 to 99.3%. It includes the factors age, stage, hemoglobin and splenic involvement [15]. However, the optimal implementation into risk allocation systems remains to be determined for both the GP and the LP-IPS.

In summary, the present analysis indicated an improved GP-based discrimination of NLPHL risk groups by dividing patients into GP ABCF vs DE. In advanced-stage disease, inferior outcomes for patients with GP DE were not diminished by intensive treatment based on BEACOPP chemotherapy. Given these results, initial presentation with GP DE might represent a possible risk factor in future risk group allocation strategies for NLPHL.

## Supplementary information

Supplemental Figure 1

Supplemental Figure 2

Supplemental Table 1

Supplemental Table 2

## Data Availability

Data can be made available upon reasonable request. Decisions regarding data sharing will be made on a case-by-case basis by the corresponding author considering data protection and other applicable regulations.
