# Supplementary material for: Refining histopathological growth pattern-based risk group discrimination in nodular lymphocyte-predominant Hodgkin lymphoma: an analysis from the German Hodgkin Study Group
Source: Leukemia. 2025 May 13;39(7):1735–43. doi: 10.1038/s41375-025-02641-3 (PMC12208872; doi:10.1038/s41375-025-02641-3)
Supplement: Supplementary file 1 — Supplemental Figure 1 [file 41375_2025_2641_MOESM1_ESM.pptx]

## Slide 1
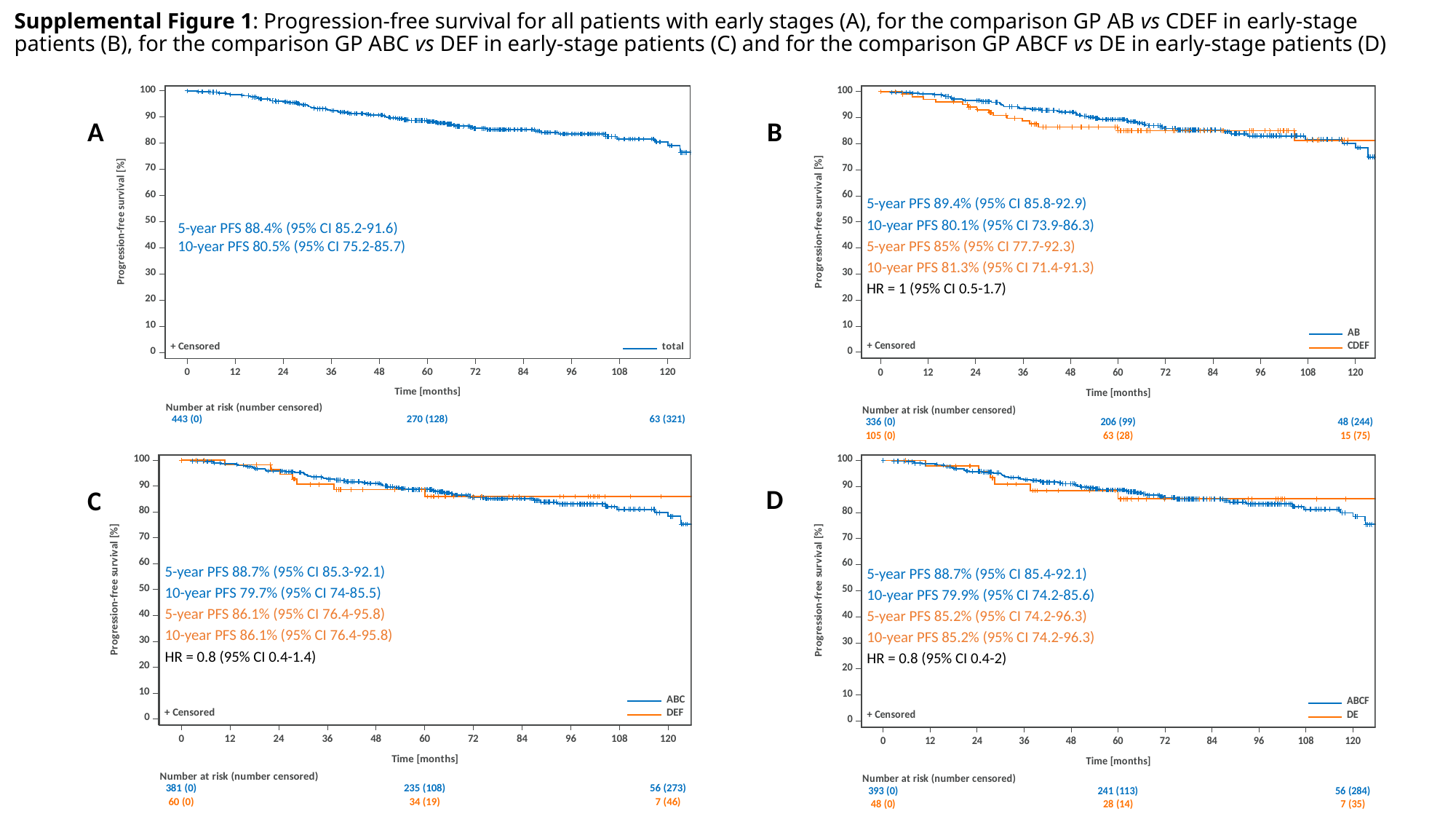

# Supplemental Figure 1: Progression-free survival for all patients with early stages (A), for the comparison GP AB vs CDEF in early-stage patients (B), for the comparison GP ABC vs DEF in early-stage patients (C) and for the comparison GP ABCF vs DE in early-stage patients (D)
A
B
5-year PFS 89.4% (95% CI 85.8-92.9)
10-year PFS 80.1% (95% CI 73.9-86.3)
5-year PFS 85% (95% CI 77.7-92.3)
10-year PFS 81.3% (95% CI 71.4-91.3)
HR = 1 (95% CI 0.5-1.7)
5-year PFS 88.4% (95% CI 85.2-91.6)
10-year PFS 80.5% (95% CI 75.2-85.7)
D
C
5-year PFS 88.7% (95% CI 85.3-92.1)
10-year PFS 79.7% (95% CI 74-85.5)
5-year PFS 86.1% (95% CI 76.4-95.8)
10-year PFS 86.1% (95% CI 76.4-95.8)
HR = 0.8 (95% CI 0.4-1.4)
5-year PFS 88.7% (95% CI 85.4-92.1)
10-year PFS 79.9% (95% CI 74.2-85.6)
5-year PFS 85.2% (95% CI 74.2-96.3)
10-year PFS 85.2% (95% CI 74.2-96.3)
HR = 0.8 (95% CI 0.4-2)
