# Supplementary material for: Refining histopathological growth pattern-based risk group discrimination in nodular lymphocyte-predominant Hodgkin lymphoma: an analysis from the German Hodgkin Study Group
Source: Leukemia. 2025 May 13;39(7):1735–43. doi: 10.1038/s41375-025-02641-3 (PMC12208872; doi:10.1038/s41375-025-02641-3)
Supplement: Supplementary file 2 — Supplemental Figure 2 [file 41375_2025_2641_MOESM2_ESM.pptx]

## Slide 1
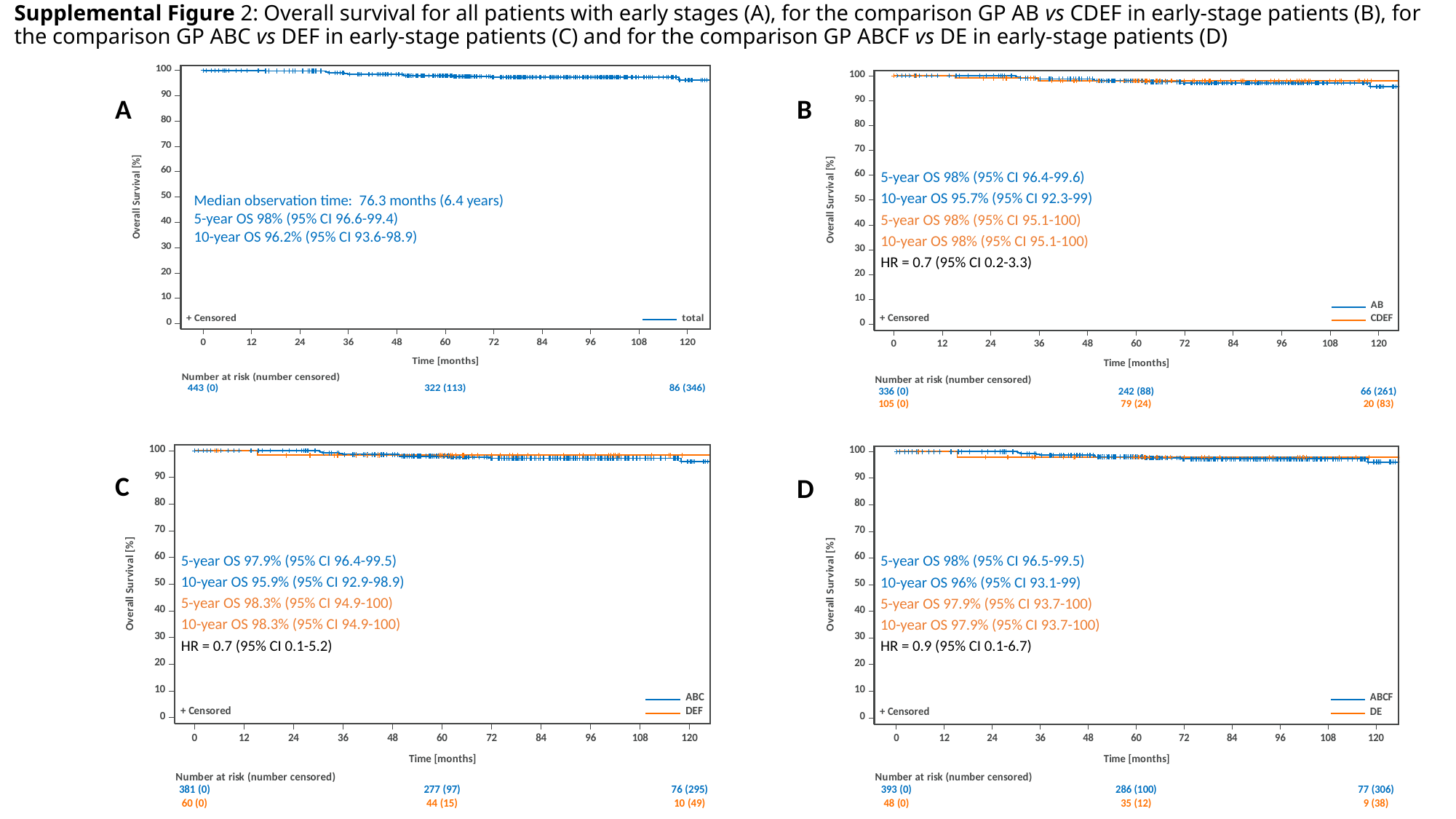

# Supplemental Figure 2: Overall survival for all patients with early stages (A), for the comparison GP AB vs CDEF in early-stage patients (B), for the comparison GP ABC vs DEF in early-stage patients (C) and for the comparison GP ABCF vs DE in early-stage patients (D)
A
B
5-year OS 98% (95% CI 96.4-99.6)
10-year OS 95.7% (95% CI 92.3-99)
5-year OS 98% (95% CI 95.1-100)
10-year OS 98% (95% CI 95.1-100)
HR = 0.7 (95% CI 0.2-3.3)
Median observation time: 76.3 months (6.4 years)
5-year OS 98% (95% CI 96.6-99.4)
10-year OS 96.2% (95% CI 93.6-98.9)
C
D
5-year OS 97.9% (95% CI 96.4-99.5)
10-year OS 95.9% (95% CI 92.9-98.9)
5-year OS 98.3% (95% CI 94.9-100)
10-year OS 98.3% (95% CI 94.9-100)
HR = 0.7 (95% CI 0.1-5.2)
5-year OS 98% (95% CI 96.5-99.5)
10-year OS 96% (95% CI 93.1-99)
5-year OS 97.9% (95% CI 93.7-100)
10-year OS 97.9% (95% CI 93.7-100)
HR = 0.9 (95% CI 0.1-6.7)
