# Supplementary material for: Refining histopathological growth pattern-based risk group discrimination in nodular lymphocyte-predominant Hodgkin lymphoma: an analysis from the German Hodgkin Study Group
Source: Leukemia. 2025 May 13;39(7):1735–43. doi: 10.1038/s41375-025-02641-3 (PMC12208872; doi:10.1038/s41375-025-02641-3)
Supplement: Supplementary file 3 — Supplemental Table 1 [file 41375_2025_2641_MOESM3_ESM.docx]

**Supplemental Table 1: Histopathological growth patterns according to different risk groups**

| **Pattern** | **Stage IA without risk factors** | **Early favorable stages** | **Early unfavorable stages** | **Advanced stages** | **Total** |
| --- | --- | --- | --- | --- | --- |
| **n (%)** | | | | | |
| **AB** | 134 (79.8) | 149 (73) | 53 (76.8) | 72 (51.4) | 408 (70.2) |
| **C** | 18 (10.7) | 20 (9.8) | 7 (10.1) | 15 (10.7) | 60 (10.3) |
| **D** | 9 (4.5) | 20 (9.8) | 4 (5.8) | 35 (25) | 68 (11.7) |
| **E** | 2 (1.2) | 10 (4.9) | 3 (4.2) | 15 (10.7) | 30 (5.2) |
| **F** | 5 (3) | 5 (2.5) | 2 (2.9) | 3 (2.1) | 15 (2.6) |
| **Total** | 168 (100) | 204 (100) | 69 (100) | 140 (100) | 581 (100) |
