# Supplementary material for: Refining histopathological growth pattern-based risk group discrimination in nodular lymphocyte-predominant Hodgkin lymphoma: an analysis from the German Hodgkin Study Group
Source: Leukemia. 2025 May 13;39(7):1735–43. doi: 10.1038/s41375-025-02641-3 (PMC12208872; doi:10.1038/s41375-025-02641-3)
Supplement: Supplementary file 4 — Supplemental Table 2 [file 41375_2025_2641_MOESM4_ESM.docx]

**Supplemental Table 2: Characteristics of second primary malignancies**

|  |  | **AB**  **(N=408)** | **CDEF**  **(N=175)** | **ABC**  **(N=468)** | **DEF**  **(N=113)** | **ABCF**  **(N=483)** | **DE**  **(N=98)** | **Total**  **(N=583)** |
| --- | --- | --- | --- | --- | --- | --- | --- | --- |
| n (%) | | | | | | | | |
| **Type of second primary malignancy** |  | 30 (7.4) | 8 (4.6) | 32 (6.8) | 6 (5.3) | 34 (7) | 4 (4.1) | 38 (6.5) |
|  | **Hematologic** | 12/30 (40) | 6/8 (75) | 14/32 (43.8) | 4/6 (66.7) | 14/34 (20) | 4/4 (100) | 18/38 (47.4) |
|  | **Solid** | 18/30 (60) | 2/8 (25) | 18/32 (56.3) | 2/6 (33.3) | 20/34 (80) | - | 20/38 (52.6) |
| **Months to second primary malignancy** | **Median (min-max)** | 39  (7-178) | 35  (10-151) | 35  (7-178) | 38  (10-151) | 37  (7-178) | 35  (33-115) | 37  (7-178) |
| **- Hematologic second malignancy** | **Median (min-max)** | 21  (9-165) | 34  (33-115) | 24  (9-165) | 37  (10-115) | 24  (9-165) | 37  (10-115) | 33  (9-165) |
| **- Solid second malignancy** | **Median (min-max)** | 43  (7-178) | 151 | 43  (7-178) | 151 | 44  (7-178) | - | 44  (7-178) |
